# Sex-related impacts on clinical outcomes after percutaneous coronary intervention

**DOI:** 10.1038/s41598-020-72296-w

**Published:** 2020-09-17

**Authors:** Hyun Woo Park, Seungbong Han, Gyung-Min Park, Soe Hee Ann, Jon Suh, Yong-Giun Kim, Seung-Whan Lee, Young-Hak Kim

**Affiliations:** 1 Department of Cardiology, SoonChunHyang University Bucheon Hospital, SoonChunHyang University College of Medicine, 170 Jomaru-ro, 14584 Bucheon, Republic of Korea; 2 Department of Applied Statistics, Gachon University, Seongnam, Republic of Korea; 3 Department of Cardiology, Regional Cardiocerebrovascular Center, Ulsan University Hospital, 877 Bangeojinsunhwando-ro, Dong-gu, 44033 Ulsan, Republic of Korea; 4 Division of Cardiology, Department of Internal Medicine, University of Ulsan College of Medicine, 877 Bangeojinsunhwando-ro, Dong-gu, 44033 Ulsan, Republic of Korea; 5 Department of Cardiology, Asan Medical Center, University of Ulsan College of Medicine, Seoul, Republic of Korea

**Keywords:** Cardiology, Interventional cardiology

## Abstract

The aim of this study is to investigate sex-related impacts on clinical outcomes after percutaneous coronary intervention (PCI). We analyzed 90,305 patients (29.0% of women) with the first episode of coronary artery disease who underwent PCI from the Korean National Health Insurance claims database between July 2013 and June 2017. Women were significantly older than men (71.5 ± 10.5 vs. 61.8 ± 11.7 years, *p* < 0.001). The study population had a median follow-up of 2.2 years (interquartile range, 1.2–3.3). In the propensity-score matched angina population (15,104 pairs), the in-hospital mortality of women was not different from men (odds ratio, 0.87; 95% confidence interval: 0.71–1.08, *p* = 0.202). However, the post-discharge mortality of women was significantly lower (hazard ratio, 0.74; 95% confidence interval: 0.69–0.80, *p* < 0.001) than that of men. In the propensity-score matched acute myocardial infarction (AMI) population (8,775 pairs), the in-hospital mortality of women was significantly higher than that of men (odds ratio, 1.19; 95% confidence interval: 1.05–1.34, *p* = 0.006). Meanwhile, there was no difference in mortality after discharge (hazard ratio, 0.98; 95% confidence interval: 0.91–1.06, *p* = 0.605). The post-discharge mortality of women was not higher than men under the contemporary PCI treatment. Altered sex-related impacts on clinical outcomes might be attributed to improved medical and procedural strategies.

## Introduction

There are sex-related differences in the clinical presentations, cardiovascular risk profiles, and therapeutic interventions of patients with cardiovascular disease; these differences are cited as reasons for worse prognosis in women after cardiovascular events^1,2^. On the other hand, in spite of a reportedly lower coronary revascularization rate in women, several observational and registry data have demonstrated that sex is not an independent predictor for worse clinical outcomes^3,4^. Moreover, percutaneous coronary intervention (PCI) with a contemporary drug-eluting stent (DES) has shown improved clinical outcomes in women and men^5,6^. Although mortality caused by coronary artery disease (CAD) has decreased substantially in recent decades due to changes in cardiovascular risk factors and improvements in therapeutic modalities^7–9^, the impacts of sex on clinical outcomes under concurrent PCI have not been fully clarified yet. Therefore, we investigated the sex-related differences in clinical outcomes using the National Health Insurance (NHI) claims database from South Korea.

## Methods

### Data sources and ethics

The NHI service is a compulsory social insurance that includes the entire Korean population. The Health Insurance Review and Assessment Service (HIRA) is a quasi-governmental organization that systematically reviews medical services and procedures on a fee-for-service basis. All NHI claims are reviewed by the HIRA. The diagnostic codes of the International Classification of Diseases, 10th Revision (ICD-10) and specific information about medications, devices, and procedures were also identified by codes from the HIRA claims database^10^. This study was approved by the local institutional review board of Ulsan University Hospital, Ulsan, Korea and no informed consent was required. Dr. Ann had full access to all the data in the study and takes responsibility for its integrity and the data analysis.

### Study cohort

We identified patients aged 18 years and older who had undergone PCI (codes M6551, M6552, M6561-4, M6571, or M6572) between July 2013 and June 2017 for the diagnosis of CAD (ICD-10 codes I20.X-I25.X). Patients who had a diagnostic code of CAD (ICD-10 codes I20.X–25.X) within 6 months of the indexed PCI day were excluded; this was done to selectively enroll the patients experiencing the first episode of CAD. Patients were classified by acute myocardial infarction (AMI) or angina pectoris and each diagnostic category was analyzed separately. AMI was defined by the discharge diagnostic codes from HIRA databases (ICD-10 codes I21.X–I22.X).

### Study variables

ICD-10 codes within 6 months of the indexed PCI day were used to identify comorbidities such as diabetes mellitus, hyperlipidemia, hypertension, congestive heart failure, arrhythmia, valvular disease, peripheral vascular disease, cerebrovascular disease, chronic pulmonary disease, moderate-to-severe liver disease, renal disease, cancer, and rheumatic diseases^10^. Anti-diabetic, anti-hypertensive, or anti-hyperlipidemic drugs were identified from medication codes in the HIRA database within 6 months of the indexed procedure. Additionally, patients who were taking anti-diabetic, anti-hypertensive, or anti-hyperlipidemic medications were considered as patients with diabetes mellitus, hypertension, and hyperlipidemia^11^. The Charlson comorbidity index was calculated based on these comorbidities^10^. Angioplasties were classified from the HIRA claims as DES (codes J5083XXX or J8083XXXX), bioresorbable vascular scaffold (BVS, code J5084XXXX), bare metal stents (BMS, codes J5231XXX, J5232XXX, or J8231XXXX), or non-stent coronary balloon angioplasty (if the codes for stents were not documented)^12^. Information regarding the cardiovascular medication during the hospitalization, including antiplatelet agents, statins, beta-blockers, angiotensin-converting enzyme inhibitors, and angiotensin receptor blockers, was also obtained.

### Clinical outcomes

The clinical endpoint of this study was all-cause mortality, which included in-hospital and post-discharge mortality. All-cause mortality was identified by all in- and outpatient HIRA claims that indicated death. Repeat coronary revascularization was identified using the procedure codes of PCI (M6551, M6552, M6561-4, M6571, or M6572) and coronary artery bypass grafting (O1641, O1642, O1647, OA641, OA642, or OA647). In patients with multiple events, the first event was considered to be the component of the composite outcome. The HIRA database was reviewed in this study through December 2017.

### Statistical analyses

We conducted statistical analyses in the angina and AMI cohorts separately. Baseline patient characteristics and comorbidities were presented as mean ± standard deviation for continuous variables and frequency (percentage, %) for categorical variables. Continuous variables between the sexes were compared using the Mann–Whitney U test, while categorical variables were compared using the chi-square or Fisher’s exact test, as appropriate. In order to obtain the sex-related effects on the in-hospital mortality, we conducted both univariate and multivariable logistic regression analyses. In the multivariable model, the possible adjustment factors were age, comorbidities, type of PCI treatment, number of stents, medications during the hospitalization, and the Charlson comorbidity index (Table 1). The final multivariable adjustment model was selected based on the backward variable selection approach. Cumulative incidence rates for the survival clinical outcomes between the sexes were estimated using the Kaplan–Meier method and were compared using the log-rank test. The multivariable adjusted Cox regression analyses were conducted with the possible adjustment factors, similarly to the multivariable logistic regression model. In addition, to reduce the impact of potential confounding variables on the sex-related effects, propensity-score matching analyses were conducted. The propensity scores were obtained non-parametrically using age, comorbidities, the type and number of stents, medications during the hospitalization, and the Charlson comorbidity index. The propensity-score matching was performed following the nearest-neighbor matching approach with a caliper size of 0.2 multiplied by the standard deviation for linearly transformed propensity scores (logit-transformation). The balance of confounding variables in the matched groups was evaluated by measuring their standardized mean differences between the sex-matched groups. All standardized mean differences for the confounding variables were less than 0.05 (5%) (Supplementary Figure 1 and 2). We also conducted the paired t-test or McNemar’s test for continuous or categorical variables to assess the covariate balancing between the sex-matched groups. In the propensity score-matched cohort, the Cox regression model, which was based on the robust standard errors and the generalized estimating equation model, was used to account for the clustering of matched pairs. We also conducted the landmark analysis (after 30 days) to compare the long-term mortality between sexes (Supplementary Table 1). All data analyses were performed using R software version 3.6.1 (R Foundation for Statistical Computing, Vienna, Austria; www.r-project.org). R “MatchIt” package was used for the propensity-score matching. Also, R packages of “survival” and “geepack” were used for the Cox regression and the generalized estimating equation model analyses. A *p*-value < 0.05 was considered statistically significant and all *p*-values were two-tailed.

**Table 1:** Characteristics of patients undergoing PCI in South Korea according to sex.

	Angina (n = 50,256)			AMI (n = 40,049)		
	Women (n = 16,344)	Men (n = 33,912)	*p*-value	Women (n = 9,810)	Men (n = 30,239)	*p*-value
**Baseline characteristics**						
Age	70.6 ± 10.3	63.0 ± 11.2	< 0.001	72.9 ± 10.8	60.6 ± 12.2	< 0.001
Diabetes	7,328 (44.8%)	12,236 (36.1%)	< 0.001	3,608 (36.8%)	7,441 (24.6%)	< 0.001
Diabetes with chronic complications	33 (0.2%)	49 (0.1%)	0.156	16 (0.2%)	22 (0.1%)	0.021
Hyperlipidemia	9,307 (56.9%)	16,656 (49.1%)	< 0.001	3,730 (38.0%)	8,209 (27.1%)	< 0.001
Hypertension	12,876 (78.8%)	21,064 (62.1%)	< 0.001	6,742 (68.7%)	12,818 (42.4%)	< 0.001
Congestive heart failure	1,867 (11.4%)	2,432 (7.2%)	< 0.001	679 (6.9%)	740 (2.4%)	< 0.001
Arrhythmia	1,677 (10.3%)	2,895 (8.5%)	< 0.001	512 (5.2%)	849 (2.8%)	< 0.001
Valvular disease	120 (0.7%)	111 (0.3%)	< 0.001	34 (0.3%)	33 (0.1%)	< 0.001
Peripheral vascular disease	2,702 (16.5%)	3,973 (11.7%)	< 0.001	1,366 (13.9%)	2,179 (7.2%)	< 0.001
Cerebrovascular disease	2,862 (17.5%)	4,688 (13.8%)	< 0.001	1,264 (12.9%)	2,166 (7.2%)	< 0.001
Chronic pulmonary disease	2,983 (18.3%)	4,782 (14.1%)	< 0.001	1,483 (15.1%)	2,971 (9.8%)	< 0.001
Moderate-to-severe liver disease	7 (0.04%)	14 (0.04%)	0.999	3 (0.03%)	12 (0.04%)	0.999
Renal disease	1,032 (6.3%)	1,970 (5.8%)	0.026	393 (4.0%)	891 (2.9%)	< 0.001
Cancer	314 (1.9%)	815 (2.4%)	0.001	166 (1.7%)	524 (1.7%)	0.823
Rheumatic disease	42 (0.3%)	53 (0.2%)	0.021	29 (0.3%)	24 (0.1%)	< 0.001
Charlson comorbidity index	1.72 ± 1.47	1.35 ± 1.44	< 0.001	1.39 ± 1.41	0.85 ± 1.19	< 0.001
**Treatment type**			< 0.001			< 0.001
DES	15,249 (93.3%)	31,493 (92.9%)		9,063 (92.4%)	28,490 (94.2%)	
BVS	63 (0.4%)	255 (0.8%)		32 (0.3%)	219 (0.7%)	
BMS	97 (0.6%)	232 (0.7%)		82 (0.8%)	223 (0.7%)	
POBA (no stent)	935 (5.7%)	1,932 (5.7%)		633 (6.5%)	1,307 (4.3%)	
Number of stents per person	1.41 ± 0.68	1.43 ± 0.71	0.006	1.44 ± 0.68	1.41 ± 0.67	0.001
**Medication during the hospitalization**						
Anti-platelet agent	16,233 (99.3%)	33,689 (99.3%)	0.770	9,769 (99.6%)	30,154 (99.7%)	0.038
Aspirin	15,236 (93.2%)	31,676 (93.4%)	0.434	9,084 (92.6%)	28,294 (93.6%)	0.001
Clopidogrel	13,391 (81.9%)	25,318 (74.7%)	< 0.001	5,838 (59.5%)	13,760 (45.5%)	< 0.001
Prasugrel	334 (2.0%)	1,672 (4.9%)	< 0.001	265 (2.7%)	2,318 (7.7%)	< 0.001
Ticagrelor	2,379 (14.6%)	6,493 (19.1%)	< 0.001	3,640 (37.1%)	14,013 (46.3%)	< 0.001
Statin	14,353 (87.8%)	30,362 (89.5%)	< 0.001	9,037 (92.1%)	28,510 (94.3%)	< 0.001
β-blocker	9,952 (60.9%)	19,881 (58.6%)	< 0.001	7,474 (76.2%)	23,954 (79.2%)	< 0.001
ACEI/ARB	9,804 (60.0%)	19,529 (57.6%)	< 0.001	6,713 (68.4%)	20,939 (69.2%)	0.132

Data are expressed as mean ± standard deviation and n (%).

ACEI, angiotensin-converting enzyme inhibitor; AMI, acute myocardial infarction; ARB, angiotensin receptor blocker; BMS, bare metal stent; BVS, bioresorbable vascular scaffold; DES, drug-eluting stent; PCI, percutaneous coronary intervention; POBA, plain old balloon angioplasty.

## Results

### Study population and characteristics

**Figure 1:**
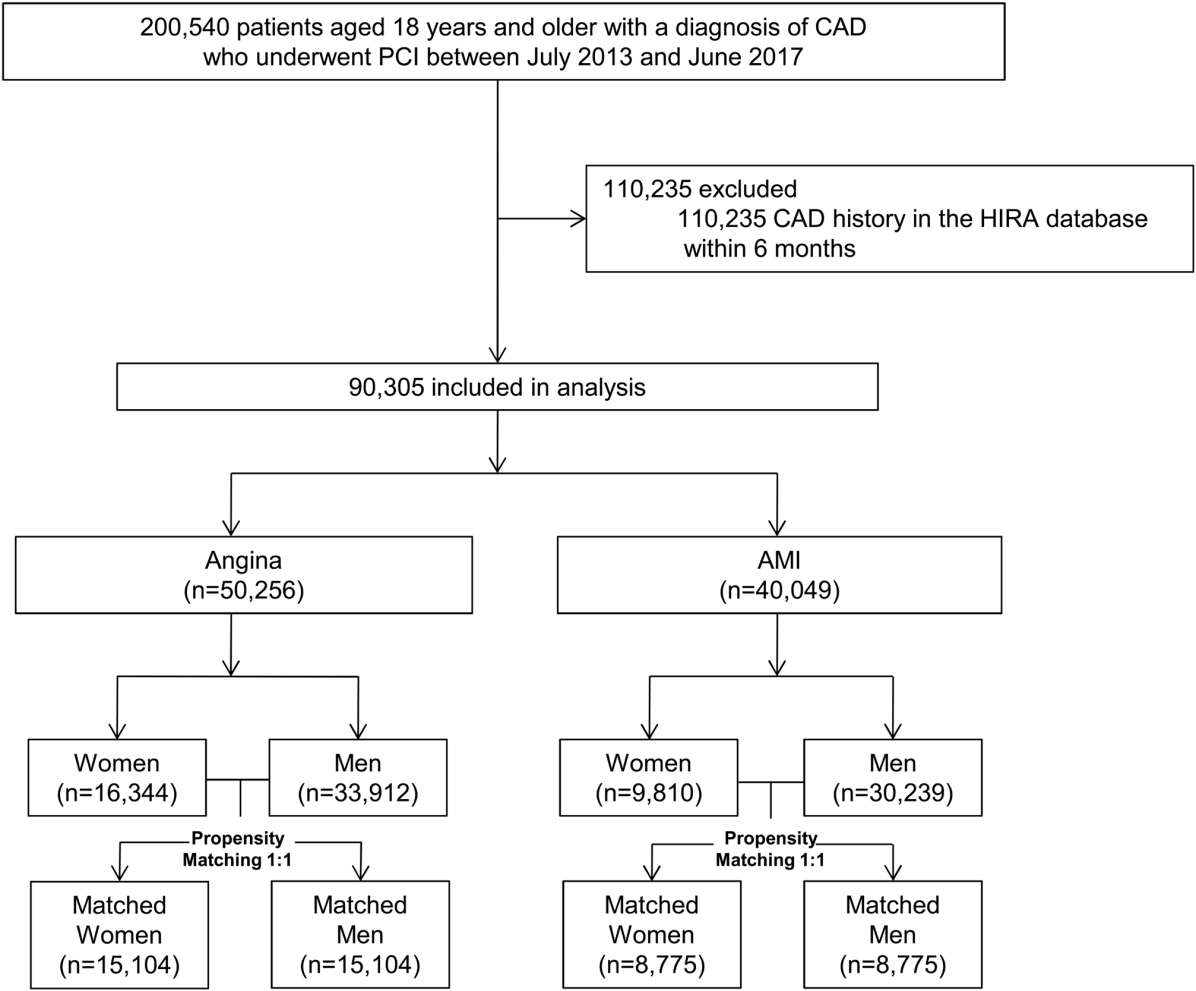
Flowchart of study design. AMI = acute myocardial infarction; CAD = coronary artery disease; HIRA = Health Insurance Review and Assessment Service; PCI = percutaneous coronary intervention.

Between July 2013 and June 2017, a total of 200,540 patients aged 18 years and older were diagnosed with CAD and underwent PCI. Among these patients, 90,305 patients with the first episode of CAD were included in the analysis (Fig. 1). During the follow-up period (median, 2.2 years; interquartile range, 1.2–3.3), the mean age of the entire study population was 64.6 ± 12.2 years and 26,154 (29.0%) were women. Diabetes mellitus, hyperlipidemia, and hypertension were observed in 30,733 (34.0%), 37,902 (42.0%), and 53,500 (59.2%) patients, respectively. A DES was implanted in 93.3%, while a BVS was used in 0.6%, and a BMS was used in 0.7%. The remaining patients undergoing PCI (5.3%) were treated by balloon angioplasty. The overall in-hospital mortality of this nationwide cohort was 2.6%. During the hospitalization, anti-platelet agents, statins, beta-blockers, and angiotensin-converting enzyme inhibitors or angiotensin receptors were prescribed to 89,845 (99.5%), 82,262 (91.1%), 61,261 (67.8%), and 56,985 patients (63.1%), respectively.

### Sex-related impacts in angina pectoris

**Figure 2:**
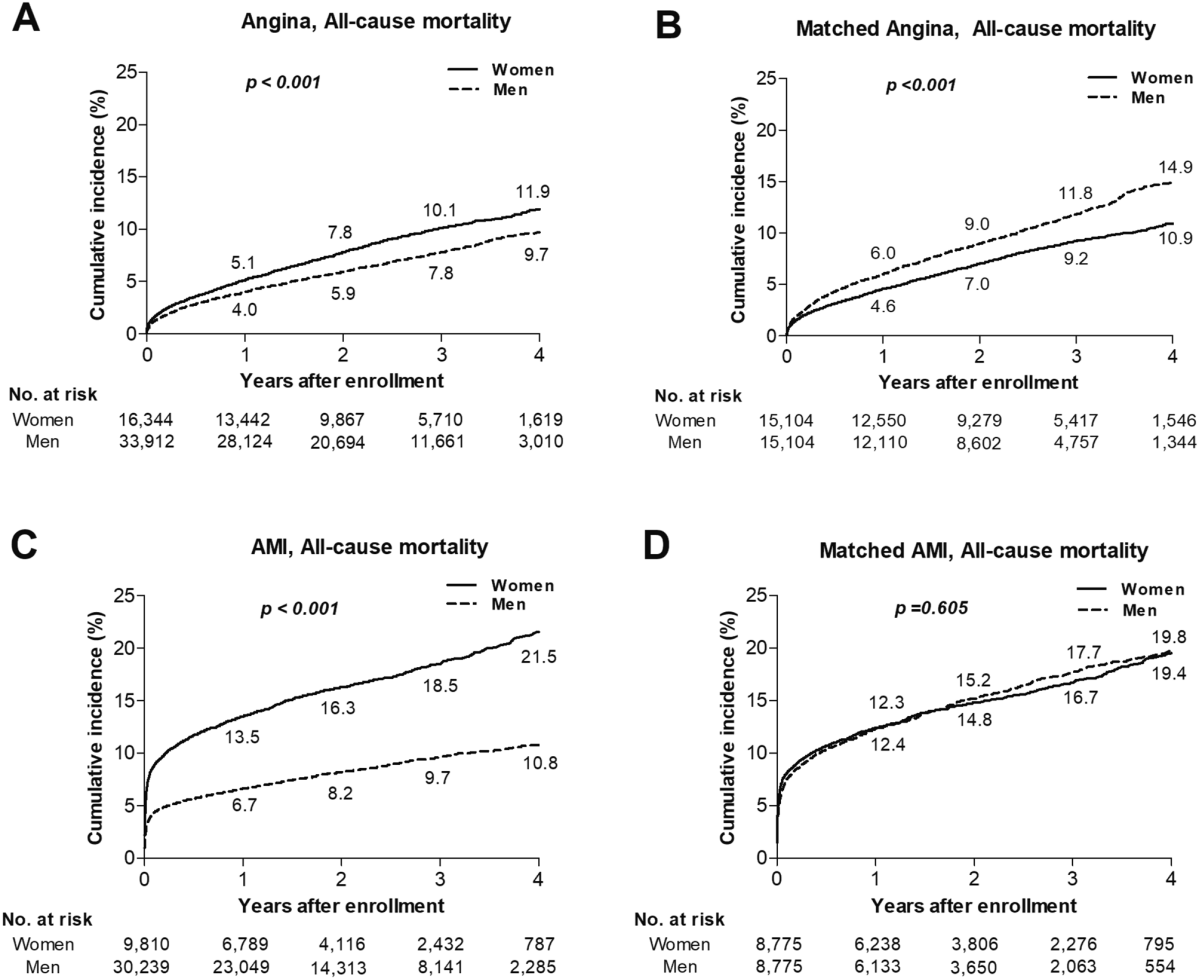
Cumulative incidence rates for all-cause mortality. The differences in cumulative incidence of all-cause mortality between sexes in angina (**A**), the matched cohort of angina (**B**), AMI (**C**) and the matched cohort of AMI (**D**). The numbers in each figure represent the cumulative incidence rates at each time point. AMI = acute myocardial infarction.

The number of patients with angina pectoris was 50,256 and 16,344 (32.5%) were women. The baseline characteristics of angina patients are shown in Table 1. The mean age of women was 7.6 years older than men and women were more likely to have major comorbidities like diabetes, hyperlipidemia, hypertension, congestive heart failure, peripheral vascular disease, cerebrovascular disease, and renal disease. As a result, the Charlson comorbidity index was significantly higher in women than men (1.72 ± 1.47 vs. 1.35 ± 1.44, *p* < 0.001). The use of BVS was more frequent and the mean number of stents per patient was higher in men. Most patients (99.3%) took antiplatelet agents during the hospitalization. However, potent P2Y12 inhibitors, such prasugrel or ticagrelor, were prescribed to more men than women. Even though the frequency of hyperlipidemia was higher in women, the prescription rate of statins was significantly lower in women. Beta-blockers and angiotensin-converting enzyme inhibitors or angiotensin receptor blockers were prescribed to more women, which reflects the higher rate of hypertension in women. In-hospital mortality was higher in women than men (1.3% vs. 0.9%, *p* < 0.001). During the follow-up period, the all-cause mortality of women was also significantly higher than that of men (8.7% vs. 6.8%, *p* < 0.001) (Fig. 2). Since the repeated revascularization rate in women was significantly lower than that in men (9.4% vs. 12.1%, *p* < 0.001), the composite of all-cause mortality and the revascularization rate were also lower in women compared to men (17.4% vs. 18.2%, *p* = 0.01). Multivariable Cox analysis shows that there was no significant difference in in-hospital mortality between women and men, while the all-cause mortality after discharge was significantly lower in women than men (Table 2). After propensity-score matching, there were 15,104 matched pairs without significant differences, in terms of covariates observed between sexes (Table 3). The in-hospital mortality rate of the matched pairs was not significantly different between women and men (odds ratio of women, 0.87; 95% confidence interval: 0.71–1.08; *p* = 0.202). The risk for post-discharge mortality was significantly lower in women than men (hazard ratio of women, 0.74; 95% confidence interval: 0.69–0.80, *p* < 0.001). Furthermore, the composite outcomes of all-cause mortality and repeat revascularization were also lower in women than men (hazard ratio of women, 0.78; 95% confidence interval: 0.74–0.82; *p* < 0.001) (Table 4).

**Table 2:** Multivariable Cox analysis for clinical outcomes.

Multivariable Cox-proportional hazards	Angina		AMI	
	Women with men			
**In-hospital mortality**	Adjusted OR (95% CI)	*p*-value	Adjusted OR (95% CI)	*p*-value
	0.93 (0.77–1.11)	0.412	1.24 (1.10–1.40)	< 0.001
**Post-discharge**	Adjusted HR (95% CI)	*p*-value	Adjusted HR (95% CI)	*p*-value
All-cause mortality	0.76 (0.71–0.82)	< 0.001	1.00 (0.93–1.07)	0.975
Death/repeat revascularization	0.79 (0.76–0.83)	< 0.001	0.98 (0.93–1.03)	0.335

Repeat revascularization includes percutaneous coronary intervention and coronary artery bypass graft.

AMI, acute myocardial infarction; HR, hazards ratio; OR, odds ratio; CI, confidence interval.

**Table 3:** Baseline characteristics in propensity-score matched pairs according to sex.

	Angina (n = 30,208)			AMI (n = 17,550)		
	Women (n = 15,104)	Men (n = 15,104)	*p*-value	Women (n = 8,775)	Men (n = 8,775)	*p*-value
**Baseline characteristics**						
Age	69.6 ± 9.9	69.2 ± 10.2	0.141	71.5 ± 10.5	71.0 ± 10.9	0.142
Diabetes mellitus	6,638 (43.9%)	6,621 (43.8%)	0.422	3,120 (35.6%)	3,106 (35.4%)	0.642
Hyperlipidemia	8,535 (56.5%)	8,440 (55.9%)	0.943	3,259 (37.1%)	3,219 (36.7%)	0.460
Hypertension	11,668 (77.3%)	11,501 (76.1%)	0.410	5,789 (66.0%)	5,682 (64.8%)	0.256
Congestive heart failure	1,574 (10.4%)	1,580 (10.5%)	0.256	478 (5.4%)	492 (5.6%)	0.710
Arrhythmia	1,537 (10.2%)	1,538 (10.2%)	0.068	417 (4.8%)	431 (4.9%)	0.456
Valvular disease	88 (0.6%)	92 (0.6%)	0.062	24 (0.3%)	25 (0.3%)	0.999
Peripheral vascular disease	2,421 (16.0%)	2,385 (15.8%)	0.436	1,148 (13.1%)	1,136 (12.9%)	0.099
Cerebrovascular disease	2,641 (17.5%)	2,678 (17.7%)	0.999	1,097 (12.5%)	1,111 (12.7%)	0.190
Chronic pulmonary disease	2,691 (17.8%)	2,706 (17.9%)	0.319	1,296 (14.8%)	1,332 (15.2%)	0.453
Moderate to severe liver disease	7 (0.05%)	6 (0.04%)	0.999	3 (0.03%)	3 (0.03%)	0.999
Renal disease	977 (6.5%)	1,000 (6.6%)	0.149	365 (4.2%)	388 (4.4%)	0.823
Cancer	309 (2.0%)	328 (2.2%)	0.569	163 (1.9%)	185 (2.1%)	0.999
Rheumatologic disease	31 (0.2%)	35 (0.2%)	0.268	18 (0.2%)	16 (0.2%)	0.999
Charlson comorbidities	1.67 ± 1.46	1.67 ± 1.52	0.217	1.32 ± 1.38	1.33 ± 1.41	0.282
**Treatment type**			0.903			0.760
DES	14,075 (93.2%)	14,077 (93.2%)		8,159 (93.0%)	8,161 (93.0%)	
BVS	61 (0.4%)	69 (0.5%)		32 (0.4%)	39 (0.4%)	
BMS	92 (0.6%)	93 (0.6%)		67 (0.8%)	73 (0.8%)	
POBA (no stent)	876 (5.8%)	865 (5.7%)		517 (5.9%)	502 (5.7%)	
Number of stents per person	1.42 ± 0.69	1.41 ± 0.69	0.495	1.45 ± 0.68	1.44 ± 0.69	0.053
**Medication during the hospitalization**						
Anti-platelet	14,996 (99.3%)	14,985 (99.2%)	0.460	8,745 (99.7%)	8,742 (99.6%)	0.440
Statin	13,274 (87.9%)	13,216 (87.5%)	0.930	8,120 (92.5%)	8,121 (92.5%)	0.646
β-blocker	9,088 (60.2%)	8,985 (59.5%)	0.814	6,716 (76.5%)	6,747 (76.9%)	0.565
ACEI/ARB	8,975 (59.4%)	8,891(58.9%)	0.841	6,019 (68.6%)	6,015 (68.5%)	0.108

Data are expressed as mean ± standard deviation and n (%).

ACEI, angiotensin-converting enzyme inhibitor; AMI, acute myocardial infarction; ARB, angiotensin receptor blocker; BMS, bare metal stent; BVS, bioresorbable vascular scaffold; DES, drug-eluting stent; PCI, percutaneous coronary intervention; POBA, plain old balloon angioplasty.

**Table 4:** Clinical outcomes in propensity-score matched pairs according to sex.

Propensity score matching	Angina (n = 15,104 pairs)		AMI (n = 8,775 pairs)	
	Women with men			
**In-hospital mortality**	OR (95% CI)	*p*-value	OR (95% CI)	*p*-value
	0.87 (0.71–1.08)	0.202	1.19 (1.05–1.34)	0.006
**Post-discharge**	HR (95% CI)	*p*-value	HR (95% CI)	*p*-value
All-cause mortality	0.74 (0.69–0.80)	< 0.001	0.98 (0.91–1.06)	0.605
Death/repeat revascularization	0.78 (0.74–0.82)	< 0.001	0.97 (0.92–1.03)	0.331

Repeat revascularization includes percutaneous coronary intervention and coronary artery bypass graft.

AMI, cute myocardial infarction; HR, hazards ratio; OR, odds ratio; CI, confidence interval.

### Sex-related impacts in acute myocardial infarction

The number of patients with AMI was 40,049 and 9,810 (24.5%) were women (Table 1). The mean age of women was 72.9 ± 10.8 years old, which was 12 years older than men. The Charlson comorbidity index was significantly higher in women than men (1.39 ± 1.41 vs. 0.85 ± 1.19, *p* < 0.001); this was likely because of the higher occurrence of comorbidities like diabetes, hyperlipidemia, hypertension, congestive heart failure, peripheral vascular disease, cerebrovascular disease, and renal disease. Although the number of stents was significantly higher in women compared to men (1.44 ± 0.68 vs. 1.41 ± 0.67, *p* < 0.001) in AMI, the rate of DES implantation was significantly lower and balloon angioplasty was higher in women. During the hospitalization, aspirin was less prescribed in women than men (99.6% vs. 99.7%, *p* = 0.038). Potent P2Y12 inhibitors were prescribed to more men, while clopidogrel was more commonly used in women with AMI. The prescription rates of statins and beta-blockers were also lower in women. However, angiotensin-converting enzyme inhibitors or angiotensin receptor blockers were prescribed to similar numbers of women and men. The in-hospital mortality rate in women was more than twice that of men (7.5% vs. 3.7%, *p* < 0.001). During the follow-up period, the all-cause mortality of women was also significantly higher than men (16.5% vs. 8.4%, *p* < 0.001). Although the rate of repeat revascularization of women was significantly lower than men (11.7% vs. 14.8%, *p* < 0.001), the composite of all-cause mortality and revascularization still showed unfavorable clinical outcomes in women (27.3% vs. 22.6%, *p* < 0.001). Multivariable Cox analysis shows that in-hospital mortality was significantly higher in women than men, however, there was no significant difference in the all-cause mortality after discharge between women and men (Table 2). After propensity-score matching, there were 8,775 matched pairs with no significant differences, in terms of covariates observed between sexes (Table 3). The in-hospital mortality of matched women was significantly higher compared to matched men (odds ratio of women, 1.19; 95% confidence interval: 1.05–1.34; *p* = 0.006). However, the post-discharge mortality rate was similar between women and men (hazard ratio of women, 0.98; 95% confidence interval: 0.91–1.06, *p* = 0.605). Furthermore, there were no significant differences for the composite outcomes of all-cause death and repeat revascularization between sexes (hazard ratio of women, 0.97; 95% confidence interval: 0.92–1.03; *p* = 0.331) (Table 4).

## Discussion

In this study, risk profiles, including older age, diabetes mellitus, hyperlipidemia, hypertension, congestive heart failure, peripheral vascular disease, cerebrovascular disease, and renal disease, were more common in women than men. Women showed higher mortality after PCI both in angina and AMI. To investigate the genuine impacts of sex on clinical outcomes, we statistically adjusted these confounding factors using multivariable Cox regression and propensity-score matching analyses. After statistical adjustments, the in-hospital mortality of women was not significantly different from that of men in the matched angina population, but still higher than men in the matched AMI population. On the other hand, during the follow-up period after hospital discharge, the risk for mortality was 26% lower for women in the matched angina population and similar between the sexes in the matched AMI population.

Until now, inconsistent registry data has been reported regarding clinical outcomes and potential sex-related differences^13–15^. Compared to previous registry data, a strength of this study is its all-inclusive design; we included the entire Korean population who underwent contemporary PCI for angina or AMI^12^. This nationwide cohort is not confined to specific participating hospitals, locations, and operators and, therefore, the generalizability of the data may be increased. Furthermore, aggressive medical treatment for secondary prevention was followed based on current guidelines, which suggest that it provides relevant clinical information for genuine sex-related impacts on clinical outcomes after PCI.

Previous studies have shown that women with ischemic heart disease have a poorer clinical prognosis compared to men. In the German PCI registry, there was no significant difference in the in-hospital mortality rate in women who underwent PCI compared to men, except ST-segment elevation myocardial infarction (STEMI) patients without cardiogenic shock^16^. Meanwhile, in STEMI patients without cardiogenic shock, women undergoing PCI had poorer clinical outcomes than men. Identifying the STEMI subgroup was not feasible in the present study and heterogeneous patients with various clinical situations might be included in our AMI cohort. The German registry included patients with a previous history of PCI and/or coronary artery bypass grafting, while our study only included patients who underwent PCI for the first time.

Recently, a large-scale meta-analysis involving 1,032,828 patients (25% of women) showed that both the in-hospital and 2-year mortality rates after PCI were lower in men than in women^17^. And nationwide cohort of 6.6 million PCI procedures in the United States showed higher in-hospital mortality and procedural complications observed in women after multivariable logistic regressions^18^. In our overall cohort of angina and AMI, the in-hospital mortality rate was also significantly higher in women, which could be attributed to the in-hospital outcomes of AMI patients. However, there was a lower post-discharge mortality rate in women, which could be attributed to the outcomes of patients with angina pectoris. Furthermore, these differences may be accredited to the adjustment for age and risk factors, such as hypertension, hyperlipidemia, diabetes, and heart failure, in our propensity-score matching analysis.

A progressive decline in the mortality rate of coronary disease has been reported for several decades by continuous efforts to modify risk factors, make diagnoses in time, and properly treat patients^7,9^. Particularly, DES implantation has enabled a higher procedural success rate in comorbid patients with complex lesions^19^; furthermore, it improved the survival rate of AMI in women and men^20^. In Korea, better clinical outcomes of women in the DES registry were previously reported^6^ and a new generation DES became the most frequently used device (93.2%) in PCI between 2011 and 2015^12^.

And women have a higher life expectancy than men, which is due to differences in deaths from injuries, lung cancer, and cardiovascular diseases, which may be related to the differential hormonal effects, genetic predisposition^21^ and smoking habits^22^. In another eastern Asian study, elderly women with STEMI showed better clinical outcomes 1 year after primary PCI than men^23^. Furthermore, there was a higher smoking rate in men than women (52.3% vs. 5.1%) in that study. Generally, the smoking rates differ by more than 10 times between men and women in Korea^24^. In this national cohort study, although information regarding smoking habits were not collected and adjusted using propensity score matching, the differences in smoking habits could contribute to the better survival rate in women with angina.

For young women, AMI with non-obstructive CAD is reportedly a worse prognostic factor^25^. Because we only included PCI patients with the first episode of CAD, this high-risk population was not included in the analyses. Finally, the changes related to aging, such as the thickening of the intima-media or stiffening with a reduction in compliance, involve the arterial wall. This is likely why there is a higher incidence of hypertension or heart failure with more complications after AMI in very elderly women^26^. The very elderly women were excluded in the propensity-score matching analysis because of the lack of men who were the same age. However, the Italian survey for elderly NSTEMI (over 75 years old) showed similar in-hospital outcomes between sexes and a better 1-year outcome in women compared to men; these findings aligned with our study outcomes^27^. Further studies should investigate the clinical outcomes after PCI in the very elderly.

This study has several limitations that should be addressed. First, it is based on administrative data from the HIRA in South Korea, which has no clinical data or medical examination results. The clinical diagnosis of acute coronary syndrome was not possible because there are no specific diagnostic codes (ICD-10) for unstable or stable angina. We were forced to categorize the study population as AMI or angina. To identify the STEMI subgroup was not feasible, suggesting heterogeneous patients with various clinical situations might be included in our AMI cohort. Different clinical presentations with various etiologies like plaque rupture, erosion and spontaneous coronary dissection could influenced on the clinical outcome. Second, while propensity-score matching in this study adjusted for measured confounding variables, it cannot adjust for unmeasured confounding variables, such as smoking, anemia, and biological factors between the sexes^28^. Third, we could not specify the cause of death but reported all-cause death using HIRA claims, and the clinical diagnosis of repeated revascularization. Finally, because this study only included a Korean population, it might not be possible to generalize our findings because of ethnic differences and different social-economic situations of other countries.

Despite the above limitations, our study is a nationwide large cohort study that can provide accurate and objective clinical outcomes of patients who underwent PCI for the first time. In Korea, most medical expenses related to PCI are obligatorily covered with the Korean NHI. Our study is also novel because it included women who underwent optimal dual antiplatelet therapy and statins at the time of PCI using almost second-generation DES. There is a greater than 50% probability that the life expectancy of women in Korea will break the 90 years old barrier by 2030, which will be the highest value in the world^29^. This nationwide cohort study might suggest the improved clinical outcomes of women under the unrestricted application of procedural and medical treatments.

## Conclusion

Under the contemporary PCI treatment in Korea, the in-hospital mortality of women, compared to men, was similar in angina pectoris but was higher in AMI. During the mid-term follow-up period, the mortality rate of women with angina was lower than men and there was not a significant difference between the sexes in AMI. Altered sex-related impacts on clinical outcomes might be attributed to improved medical and procedural strategies. Further studies are required to ascertain the mechanisms of sex-related differences in cardiovascular events.

## Supplementary information

Supplementary Information.

## Abbreviations

AMI: Acute myocardial infarction
BMS: Bare metal stent
BVS: Bioresorbable vascular scaffold
CAD: Coronary artery disease
DES: Drug-eluting stent
HIRA: The Health Insurance Review and Assessment Service
ICD-10: International Classification of Diseases, 10th Revision
NHI: National Health Insurance
PCI: Percutaneous coronary intervention
STEMI: ST-segment elevation myocardial infarction
