# Supplementary material for: Sex-related impacts on clinical outcomes after percutaneous coronary intervention
Source: Sci Rep. 2020 Sep 17;10:15262. doi: 10.1038/s41598-020-72296-w (PMC7498594; doi:10.1038/s41598-020-72296-w)
Supplement: Supplementary file 1 — Supplementary Information. [file 41598_2020_72296_MOESM1_ESM.docx]

**Sex-related impacts on clinical outcomes after percutaneous coronary intervention**

Park et al Sex-related impacts after PCI

Hyun Woo Park, MD^a^, Seungbong Han, PhD^b^, Gyung-Min Park, MD, PhD^c, d^, Soe Hee Ann, MD, PhD*^c, d^, Jon Suh, MD, PhD*^a^, Yong-Giun Kim, MD, PhD^c, d^, Seung-Whan Lee, MD, PhD^e^, Young-Hak Kim, MD, PhD^e^

^a^ Department of Cardiology, SoonChunHyang University Bucheon Hospital, SoonChunHyang University College of Medicine, Bucheon, Korea; ^b^Department of Applied Statistics, Gachon University, Seongnam, Korea; ^c^Department of Cardiology, Regional Cardiocerebrovascular Center, Ulsan University Hospital, Ulsan, Republic of Korea; ^d^Division of Cardiology, Department of Internal Medicine, University of Ulsan College of Medicine, Ulsan, Korea; ^e^Department of Cardiology, Asan Medical Center, University of Ulsan College of Medicine, Seoul, Korea.

**Corresponding Authors:**

* Soe Hee Ann, MD, PhD, Department of Cardiology, Regional Cardiocerebrovascular Center, Ulsan University Hospital, Ulsan, Republic of Korea; Division of Cardiology, Department of Internal Medicine, University of Ulsan College of Medicine, Ulsan, Korea, 877 Bangeojinsunhwando-ro, Dong-gu, Ulsan, Korea, 44033, Phone: +82-52-250-8982, Fax: +82-52-250-7048, E-mail: givingtaxi@naver.com

*Jon Suh, MD, PhD, Department of Cardiology, SoonChunHyang University Bucheon Hospital, SoonChunHyang University College of Medicine, 170 Jomaru-ro, Bucheon, Korea, 14584, Phone: +82-32-621-5867, Fax: +82-32-621-5016, E-mail: Immanuel@schmc.ac.kr

**SUPPLEMENTAL MATERIAL**

**Supplementary Table 1.** Long-term clinical outcome after landmark, 30 days

| **Propensity Score matching** | **Angina (n=14,700 pairs)** | | **AMI (n=7,847 pairs)** | |
| --- | --- | --- | --- | --- |
|  | **Women with men** | | | |
| **After Landmark of 30 days** | **HR (95% CI)** | **p-value** | **HR (95% CI)** | **p-value** |
| All-cause mortality | 0.72 (0.66-0.78) | <0.001 | 0.83 (0.75-0.93) | <0.001 |
| Death/Repeat revascularization | 0.77 (0.73-0.81) | <0.001 | 0.91 (0.85-0.98) | 0.009 |

Repeat revascularization includes percutaneous coronary intervention and coronary artery bypass graft. AMI = acute myocardial infarction; HR = hazards ratio; OR = odds ratio; CI = confidence interval

**Supplementary Figure 1.**


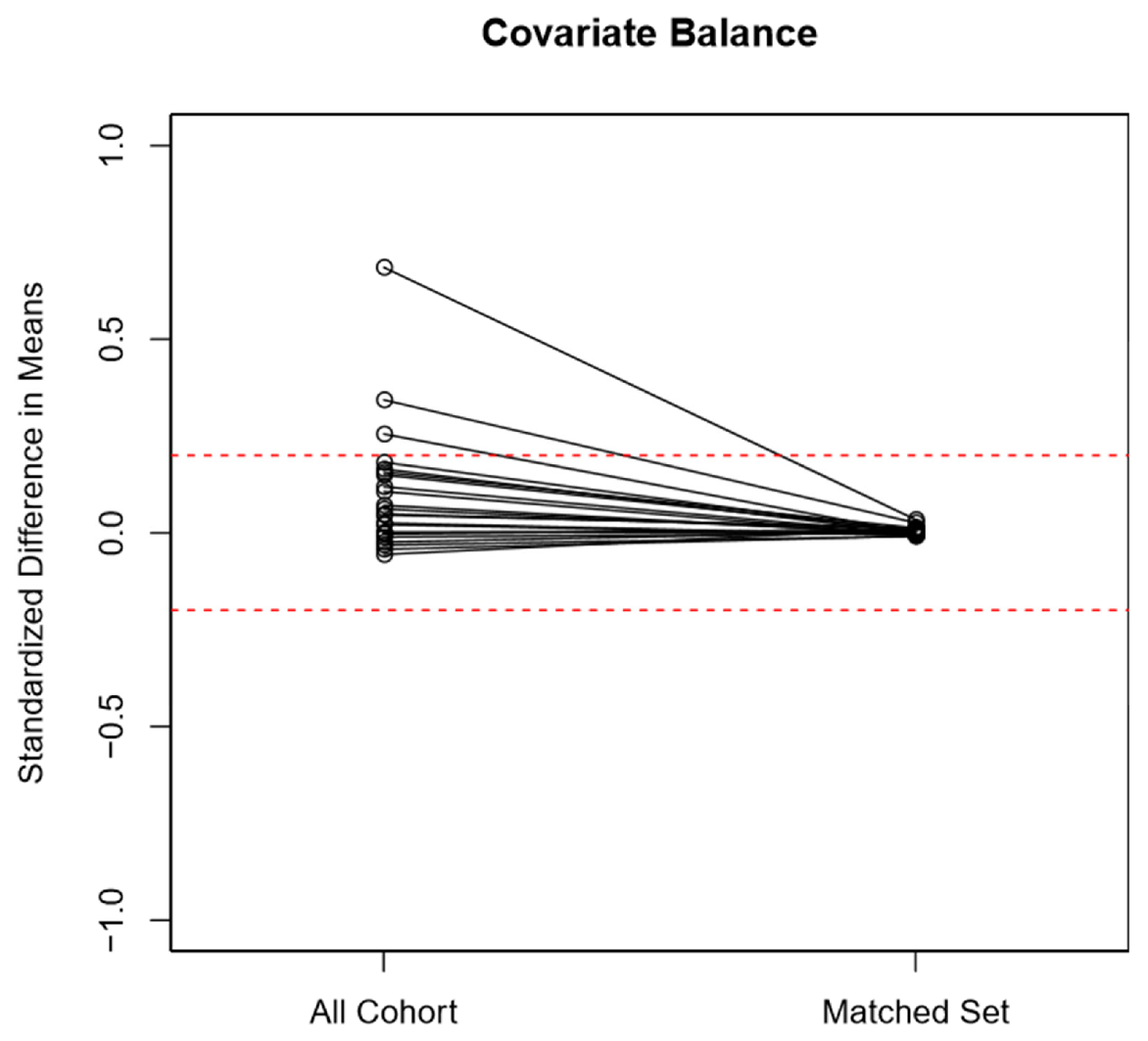


**Supplementary Figure 1.** Covariate balance in propensity-score matched patients with angina pectoris.

**Supplementary Figure 2.**


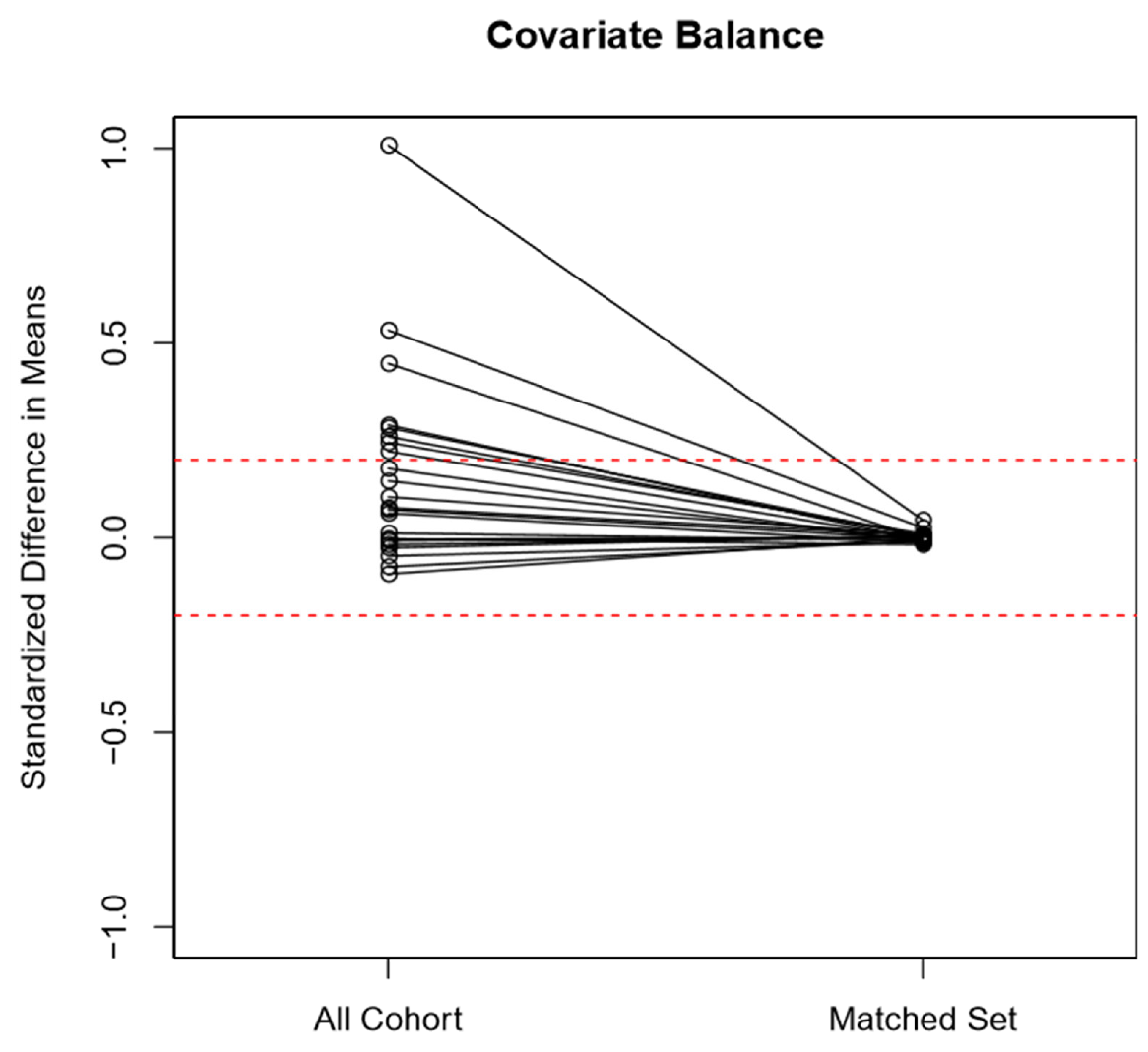


**Supplementary Figure 2.** Covariate balance in propensity-score matched patients with acute myocardial infarction.
